# Enhanced recovery after surgery program alleviates neutrophil-to-lymphocyte ratio and platelet-to-lymphocyte ratio in patients undergoing gynecological surgery

**DOI:** 10.3389/fmed.2023.1057923

**Published:** 2023-04-17

**Authors:** Naidong Xing, Hongyan Wang, Yan Huang, Jin Peng

**Affiliations:** ^1^Department of Urology, Qilu Hospital of Shandong University, Jinan, Shandong, China; ^2^Department of Obstetrics and Gynecology, Qilu Hospital of Shandong University, Jinan, Shandong, China

**Keywords:** ERAS, gynecological surgery, platelet-to-lymphocyte ratio, neutrophil-to-lymphocyte ratio, systemic inflammatory response

## Abstract

**Background:**

To evaluate the efficacy of the enhanced recovery after surgery (ERAS) programs on the systemic inflammatory response (SIR) of patients following gynecological surgery, a randomized controlled trial was performed to compare the ERAS programs with the conventional perioperative care programs. Furthermore, novel SIR markers could be identified to evaluate the ERAS programs of gynecological surgery.

**Methods:**

Patients undergoing gynecological surgery were randomly allocated to either the ERAS group or the conventional group. The correlations between the elements of ERAS protocols and SIR markers following gynecological surgery were evaluated.

**Results:**

A total of 340 patients who underwent gynecological surgery were enrolled (ERAS = 170; conventional = 170). First, we identified whether the ERAS programs after gynecological surgery reduced the perioperative difference between neutrophil-to-lymphocyte ratio (NLR) and platelet-to-lymphocyte ratio (PLR). Interestingly, first flatus time postoperatively, visual analog scale (VAS) score of patients was positively correlated with the perioperative difference NLR or PLR. Moreover, we discovered that the perioperative difference NLR or PLR was correlated with elements of ERAS protocol, including first sips of water, first semifluid diet postoperatively, pelvic drain duration, and out-of-bed time of patients.

**Conclusion:**

We originally reveal that certain elements of ERAS programs alleviated SIR to operation. The implementation of ERAS programs enhances postoperative recovery after gynecological surgery *via* improving system inflammatory status. NLR or PLR could be the novel and inexpensive marker to assess ERAS programs in gynecological surgery.

**Clinical trial registration:**ClinicalTrials.gov, identifier, NCT03629626.

## Background

Enhanced recovery after surgery (ERAS) is a multimodal perioperative protocol. Its feasibility and benefits on perioperative care have been widely reported in patients undergoing colorectal surgery, gastrointestinal surgery, urological surgery, lung surgery, hepatobiliary, pancreatic surgery, and gynecological surgery (1–7). It has been reported that ERAS can result in shorter recovery times, including better patient outcomes, less opioid utilization, less postoperative nausea and vomiting (PONV), and shorter hospital stays for gynecological surgery (8–12). Nevertheless, the ERAS impact on the systemic inflammatory response (SIR) to surgery has not yet been clearly understood (13). Neutrophils and macrophages of the innate immune system are activated by releasing proinflammatory factors such as interleukin-1 and interleukin-6 and tumor necrosis factor alpha following the cell response to surgical injury. Meanwhile, the levels of circulating acute-phase proteins, including C-reactive protein, albumin, ferritin, transferrin, and fibrinogen, are modulated by proinflammatory factors (14). The SIR features changes in relative levels of circulating white blood cells (WBCs), neutrophils, and relative lymphocytes (15). As a direct consequence of tissue trauma, interleukin-6 is synthesized locally and stimulates C-reactive protein and the fibrinogen synthesis in scar tissue growth (16). However, the blood subtype is more common and less expensive, which could be used as a clinical marker of gynecological surgical trauma in patients (17). Thus, the SIR to surgical trauma also can be easily monitored through the analyses of blood subtypes in the bloodstream, such as neutrophil-to-lymphocyte ratio (NLR), platelet-to-lymphocyte ratio (PLR), or monocyte-to-lymphocyte ratio (MLR) (18). The neutrophil-to-lymphocyte ratio (NLR) is related to clinical outcomes in patients with acute cerebral hemorrhage (19). Otherwise, a high neutrophil-to-lymphocyte ratio (NLR) or platelet-to-lymphocyte ratio (PLR) is associated with an adverse overall survival in many solid tumors (20–26). Available evidence does not show whether ERAS programs allow a measurable systemic inflammatory response (SIR) reduction in gynecological surgery. Thus, we investigated the application of ERAS principles to gynecological surgery in a prospective randomized control trial, in order to better understand the effectiveness of ERAS programs in minimizing the systemic inflammatory response, which would finally lead to shorter recovery time for patients and shorter hospital stays.

We assumed that ERAS programs would have a role in attenuating systemic inflammatory response (SIR) after gynecologic surgery. First, we demonstrated a measurable marker of SIR for ERAS following gynecological surgery, which could be a novel marker to estimate the implementation of an ERAS program after gynecologic surgery.

## Methods

After obtaining informed consent, patients who were diagnosed with gynecological benign diseases, including myoma, adenomyosis, endometrial dysplasia, or benign ovarian tumors, were eligible for enrollment. Exclusion criteria were history of constipation and American Society of Anesthesiologists risk ≥4 (27). A total of 340 patients were required to evaluate surgical procedures and perioperative care. This study was designed as a prospective, randomized control trial with a follow-up period of 6 months. Patients were randomly assigned using block randomization on a 1:1 basis to either the ERAS or the conventional group. The study was approved by the institutional research board committee of the institution in line with the STROCSS criteria (28). The trial was registered on ClinicalTrials.gov (NCT03629626).

Enhanced recovery after surgery programs are given in Table 1. Before admission, the ERAS patients were given comprehensive preoperative education. Instead of bowel preparation, a clear liquid diet was followed for 1–3 days before surgery. Meanwhile, ERAS patients were also allowed to fast up to 6 h before surgery and intake of oral carbohydrate solution (500 mL, carbohydrate 2.5%) up to 2 h before surgery. Intraoperatively, opioid IV at the discretion of an anesthesiologist was supplemented with fentanyl. After incision closure, bupivacaine was injected in transabdominal surgery. Non-steroidal anti-inflammatory drugs (NSAIDs) (50 mg intravenous flurbiprofen axetil b.i.d. for 3 days) on the day of surgery and postoperative days (POD) 1–2. The conventional group was given intravenous patient-controlled analgesia (IV PCA) mostly composed of opioid analgesics, such as fentanyl and morphine. Low-molecular-weight heparin (LMWH) and compression stockings are used as thromboprophylaxis in an ERAS setting.

**Table 1:** Enhanced recovery pathway.

Groups	ERAS
Before admission	Preoperative education operative risk assessment
Preoperative	Eliminate bowel preparation
	1–3 days fluid diet before surgery
	Fasting up to 6 h before surgery; Oral carbohydrate solution (500 mL, Carbohydrate 2.5%) up to 2 h before surgery
Intraoperative	Insertion of Foley catheter
	Antiembolic stockings
	Maintain intraoperative euvolemia: Decrease crystalloid administration
	Opioid IV at discretion of anesthesiologist supplemented with fentanyl, After incision closure: injection with bupivacaine in transabdominal surgery
Postoperative	Evening of surgery: out of bed greater than 20 min; Day after surgery and until discharge: out of bed greater than 2 h
	Patient encouraged to start drink water 2 h after surgery; Semifluid diet in POD1; general diet in POD2
	Chewing gum 24 h after surgery
	Fluid restriction (1–2 l) after surgery in POD0
	LMWH injection and antiembolic stockings
	Foley removal as early as possible
	Drain removal as early as possible
	NSAIDs for analgesia

LMWH, low-molecular-weight heparin; POD, postoperative day; IV PCA, intravenous patient-controlled analgesia.

Patients in the ERAS group were encouraged to start drinking water 2 h after surgery, chew gum 24 h after surgery, begin a semifluid diet in POD1, and returned to the general diet in POD2. Out of bed time greater than 20 min was encouraged on the day of surgery for the ERAS group and out of bed time greater than 2 h each day was encouraged after surgery until discharge. Foley and drain removal are recommended in patients in the ERAS groups as early as possible. The ERAS principles of maintenance of euvolemia and prophylactic antithrombotic were emphasized in the perioperative period. The systemic inflammatory response (SIR), hospital stay, and hospital cost were investigated. Surgical field exposure, the day of first flatus, postoperative nausea and vomiting (PONV), maximum pain score by the visual analog scale (VAS), postoperative complication, readmission rate, and re-operation rate also were demonstrated.

Patient baseline data, along with perioperative characteristics, are shown in Table 2. The time from the beginning to the end of the operation (operative time) was calculated. Gynecologic benign diseases including myoma, adenomyosis, endometrial dysplasia, and benign ovarian tumor were identified. Surgical procedure types including hysteromyomectomy, adenomyomectomy, resection of benign ovarian tumor, and hysterectomy were stated. Both laparoscopy cases and open cases are included since laparoscopic surgery is not appropriate in some circumstances, such as hyperuteri and oversize ovarian mass. To investigate the impact of ERAS on the surgical procedure, surgical field exposure was calculated as good (without intestinal distension), medium (with mild intestinal distension), and bad (with severe intestinal distension). Two experienced surgeons were involved in this study.

**Table 2:** Patient baseline data and perioperative characteristics.

	ERAS (*n* = 170)	Conventional (*n* = 170)	*p* value
**Demographics**			
Age	43.55 ± 11.04	45.51 ± 11.82	0.155
BMI (kg/m^2^)	24.91 ± 4.51	24.53 ± 4.08	0.186
Diabetes	19 (11.2)	20 (11.8)	0.236
Hypertension	7 (4.1)	9 (5.3)	0.712
Semifluid diet before surgery (days)	1.05 ± 0.37	/	
**Operative data**			
Operative time (min)	74.74 ± 28.34	79.15 ± 31.09	0.179
Laparoscopy cases	124 (72.9)	122 (71.8)	0.219
Gynecologic disease			0.529
Myoma	73 (42.94)	76 (44.71)	
Adenomyosis	25 (14.71)	18 (10.59)	
Endometrial dysplasia	15 (8.82)	20 (11.76)	
Benign ovarian tumor	57 (33.53)	56 (32.94)	
Surgical procedure			0.531
Hysteromyomectomy /adenomyomectomy	35 (20.59)	26 (15.29)	
Resection of benign ovarian tumor	34 (20)	34 (20)	
Hysterectomy	101 (59.41)	110 (64.71)	
Postoperative course			
sips of water (hours)	5.66 ± 1.25	16.24 ± 5.75	0.001
Semifluid diet (hours)	23.86 ± 1.29	38.54 ± 11.76	0.001
General diet (hours)	63.25 ± 12.16	88.80 ± 11.03	0.001
IV fluid administration in POD0 (mL)	1694.18 ± 519.25	2592.68 ± 743.21	0.001
IV fluid administration in POD1 (mL)	1161.76 ± 305.97	2163.79 ± 417.81	0.001
IV fluid administration in POD2 (mL)	703.53 ± 482.06	1628.18 ± 555.48	0.001
Out of bed time in POD0 (min)	30.65 ± 5.54	1.89 ± 5.22	0.001
Out of bed time in POD1 (min)	138.35 ± 14.46	30.47 ± 18.03	0.001
Urinary catheter duration (days)	1.75 ± 1.49	2.68 ± 0.46	0.032
Pelvic drain duration (days)	0.44 ± 0.49	2.92 ± 0.27	0.021
Complications			0.165
Ileus	1 (0.6)	1 (0.6)	
Wound infection	0 (0)	1 (0.6)	
Re-operation (%)	0 (0)	0 (0)	0.998
Readmission (%)	2 (1.2)	3 (1.8)	0.469

Data are *n* (%) or mean ± standard deviation; IV, intravenous; POD, postoperative day.

Peripheral complete blood samples were collected preoperatively and postoperatively. Different subtypes between postoperative and preoperative, including white blood cell (WBC), platelet, neutrophil, monocyte, lymphocyte, NLR, PLR, and MLR, were assessed. NLR was provided by the ratio between the absolute count of neutrophils and the absolute count of lymphocytes. PLR was calculated by dividing the absolute number of platelets by the absolute number of lymphocytes. MLR was calculated by dividing the absolute number of monocytes by the absolute number of lymphocytes.

Statistical analyses were performed using SPSS 25.0 and GraphPad Prism. Univariate analysis was used to compare the patient baseline data and operative characteristics between the two cohorts. For continuous variables, *t*-test or Mann–Whitney U-test was used, and for categorical variables, *t*-test or Fischer’s exact test was performed. Linear regression and scatter diagram showed the correlation between the first flatus or VAS and SIR. Pearson’s correlation coefficient for normally distributed data and Spearman’s correlation coefficient for non-normally distributed data were calculated between elements of the ERAS program and SIR. A value of p of <0.05 was considered statistically significant for all statistical comparisons.

## Results

**Figure 1:**
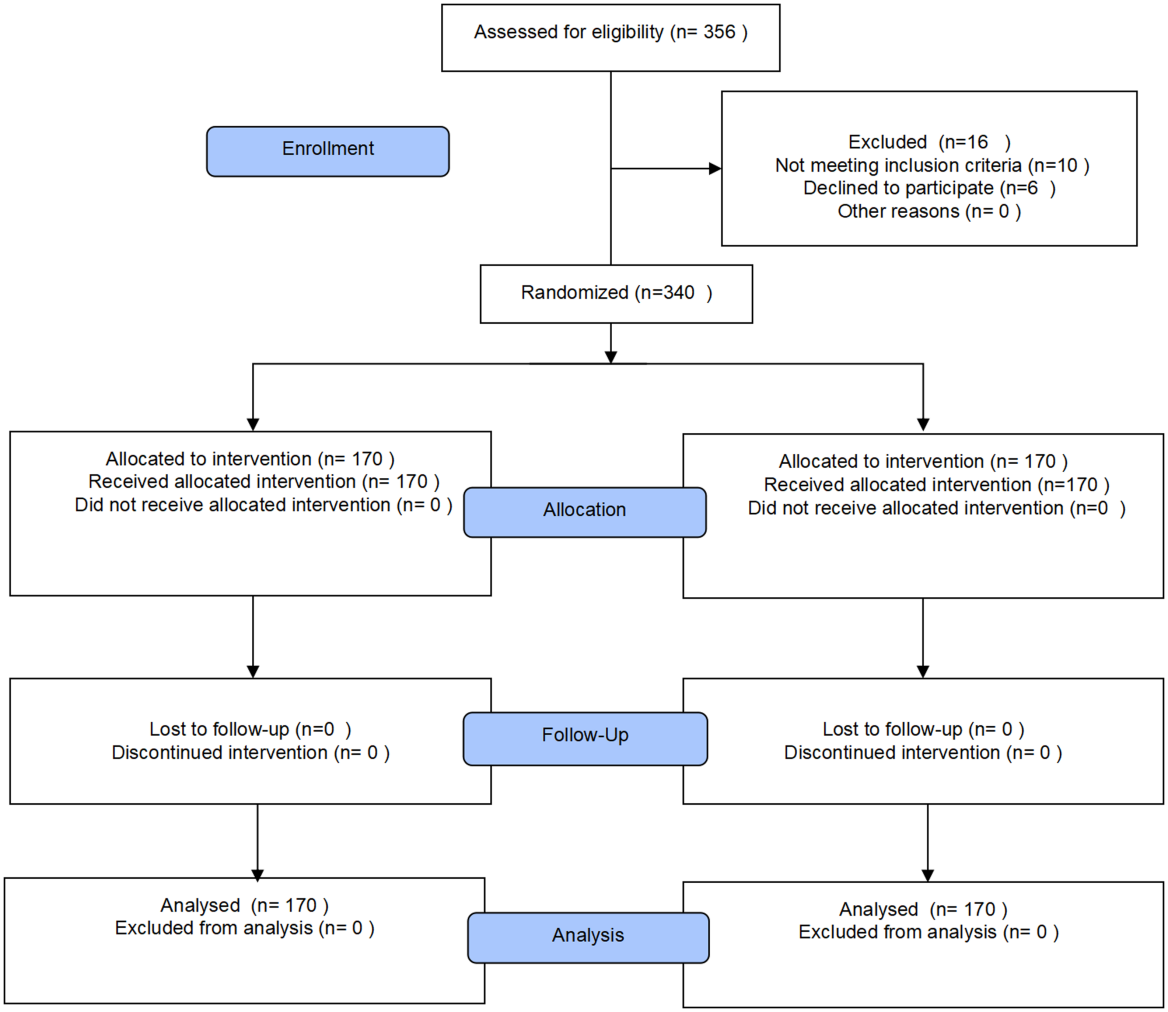
CONSORT flow diagram.

Patients undergoing gynecological surgery from September 2018 to September 2019 were included in this study. Ten patients were excluded because of constipation and ASA status, and six patients refused to proceed with the study. Finally, 170 patients were divided into the ERAS group, and 170 patients were divided into the conventional group (Figure 1). All patients were followed up postoperatively for up to 6 months. There was no statistical difference in the demographic characteristics of patients including age, body mass index (BMI), diabetes, hypertension rate, types of gynecologic disease, and surgical procedure, showing well randomization between the two groups (Table 2). The mean operative time was 74.74 ± 28.34 min in the ERAS group and 79.15 ± 31.09 min in the conventional group (*p* = 0.179, Table 2). No significant difference was shown in the laparoscopy rate between the two groups (*p* = 0.219, Table 2). The data of first sips of water, semifluid diet, and general diet were all significantly higher, while fluid administration was smaller and out-of-bed time was longer in the ERAS groups in accordance with the given protocol (Table 2). Urinary catheter and pelvic drain duration significantly reduced as required (Table 2).

**Figure 2:**
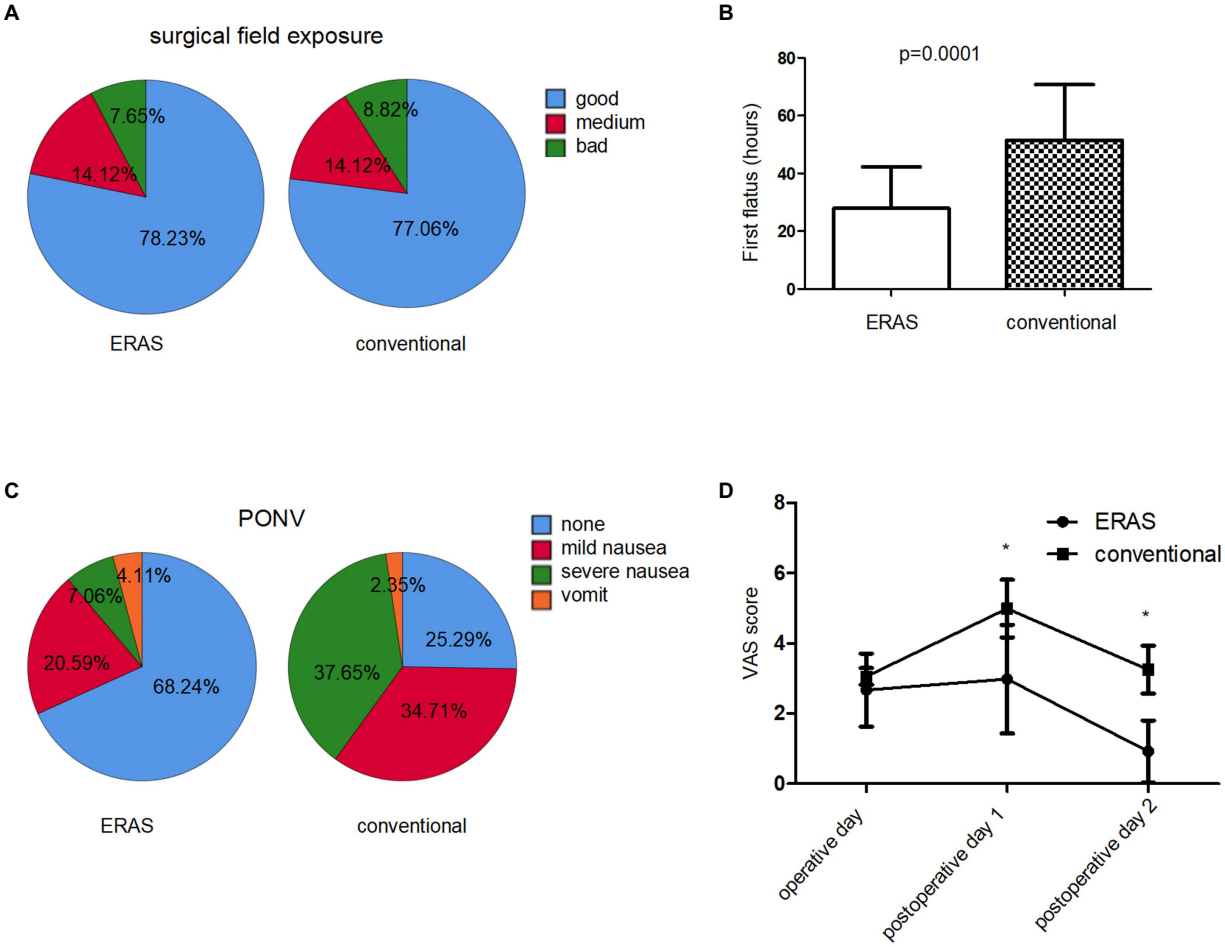
Postoperative results in ERAS and conventional group. **(A)** Surgical field exposure. **(B)** First flatus time postoperatively (hours). **(C)** Postoperative nausea and vomiting (PONV) rate after surgery. **(D)** Maximum pain (VAS) score on operative and postoperative days.

According to our observation, the ERAS protocol did not affect the surgical field exposure (*p* = 0.322, Figure 2A). Thus, the ERAS pathway had no impact on the surgical procedure, which certain surgeons worried about that, especially by eliminating bowel preparation and short fasting time. ERAS protocol may reduce the intestinal wall edema and affect the status of bowel recovery and then lead to earlier flatus postoperatively. As expected, the day of the first flatus postoperatively was faster in the ERAS group compared with the conventional group (*p* = 0.0001, Figure 2B). Moreover, patients in the ERAS group experienced significantly less postoperative nausea and vomiting (PONV) (74.71% in the conventional group compared with 31.76% in ERAS group, *p* = 0.021, Figure 2C). Despite a significant reduction in opioid, there was no change in pain scores on an operative day between the two groups (*p* = 0.612, Figure 2D). However, maximum pain score obtained by the VAS scale in ERAS group was significantly lower from postoperative day 1 to postoperative day 2 (*p* = 0.001, Figure 2D).

Patients obviously benefited from ERAS protocols; furthermore, 30-day rates of complications and re-operation did not differ between the two groups (Table 2). The postoperative readmission rate for 6 months after discharge was not different between groups (Table 2). Unsurprisingly, among hysterectomy, resection of ovarian tumors, or hysteromyomectomy/adenomyomectomy, ERAS resulted in a reduction in the total length of stay and postoperative length of stay compared with the conventional group (*p* = 0.0001, Table 3). The reduction in length of stay was accompanied by total hospital cost savings of 2000RMB per patient (*p* = 0.0001, Table 3).

**Table 3:** Recovery time and cost between ERAS and conventional cases.

	ERAS	Conventional	value of *p*
**Hysteromyomectomy/Adenomyomectomy**	(*n* = 35)	(*n* = 26)	
Postoperative length of stay	4.43 ± 1.17	4.96 ± 1.82	0.0001
Total length of stay	7.20 ± 1.55	7.92 ± 2.61	0.0001
Total hospital cost	23105.26 ± 3840.93	24589.28 ± 6398.83	0.0001
**Resection of ovarian tumor**	(*n* = 34)	(*n* = 34)	
Postoperative length of stay	3.18 ± 0.87	3.97 ± 1.38	0.0001
Total length of stay	5.44 ± 1.11	6.67 ± 1.97	0.0001
Total hospital cost	19437.24 ± 3033.73	20948.84 ± 3974.27	0.0001
**Hysterectomy**	(*n* = 101)	(*n* = 110)	
Postoperative length of stay	4.14 ± 1.21	5.02 ± 1.75	0.0001
Total length of stay	7.09 ± 1.41	8.92 ± 3.05	0.0001
Total hospital cost	25716.27 ± 5500.35	27480.13 ± 5727.56	0.0001
**Total**	(*n* = 170)	(*n* = 170)	
Postoperative length of stay	4.01 ± 1.21	4.80 ± 1.73	0.0001
Total length of stay	6.78 ± 1.53	8.31 ± 2.93	0.0001
Total hospital cost	23922.90 ± 5364.27	25731.74 ± 6090.33	0.0001

**Figure 3:**
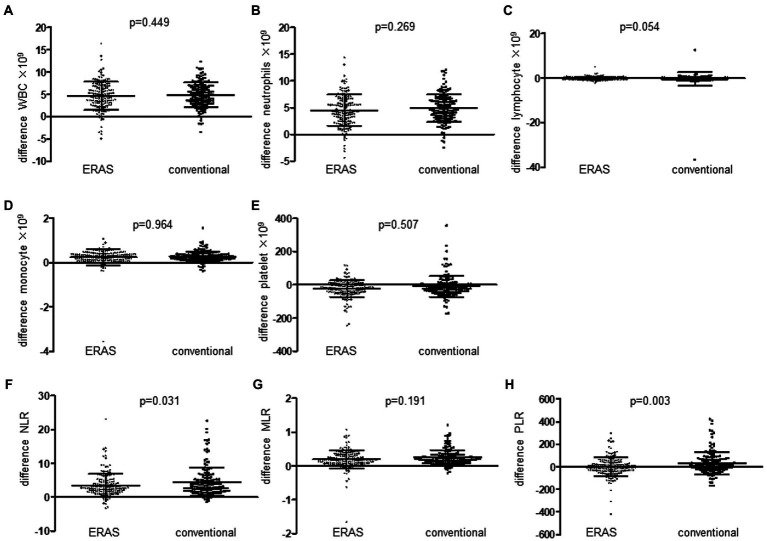
Comparison of patients following gynecologic surgery with ERAS programs and patients with conventional programs, in terms of the difference between preoperative and postoperative systemic inflammation response marker, WBC counts **(A)**, neutrophil counts **(B)**, lymphocyte counts **(C)**, monocyte counts **(D)**, platelet counts **(E)**, neutrophil-to-lymphocyte ratio (NLR) **(F)**, monocyte-to-lymphocyte ratio (MLR) **(G)**, and platelet-to-lymphocyte ratio (PLR) **(H)**.

**Figure 4:**
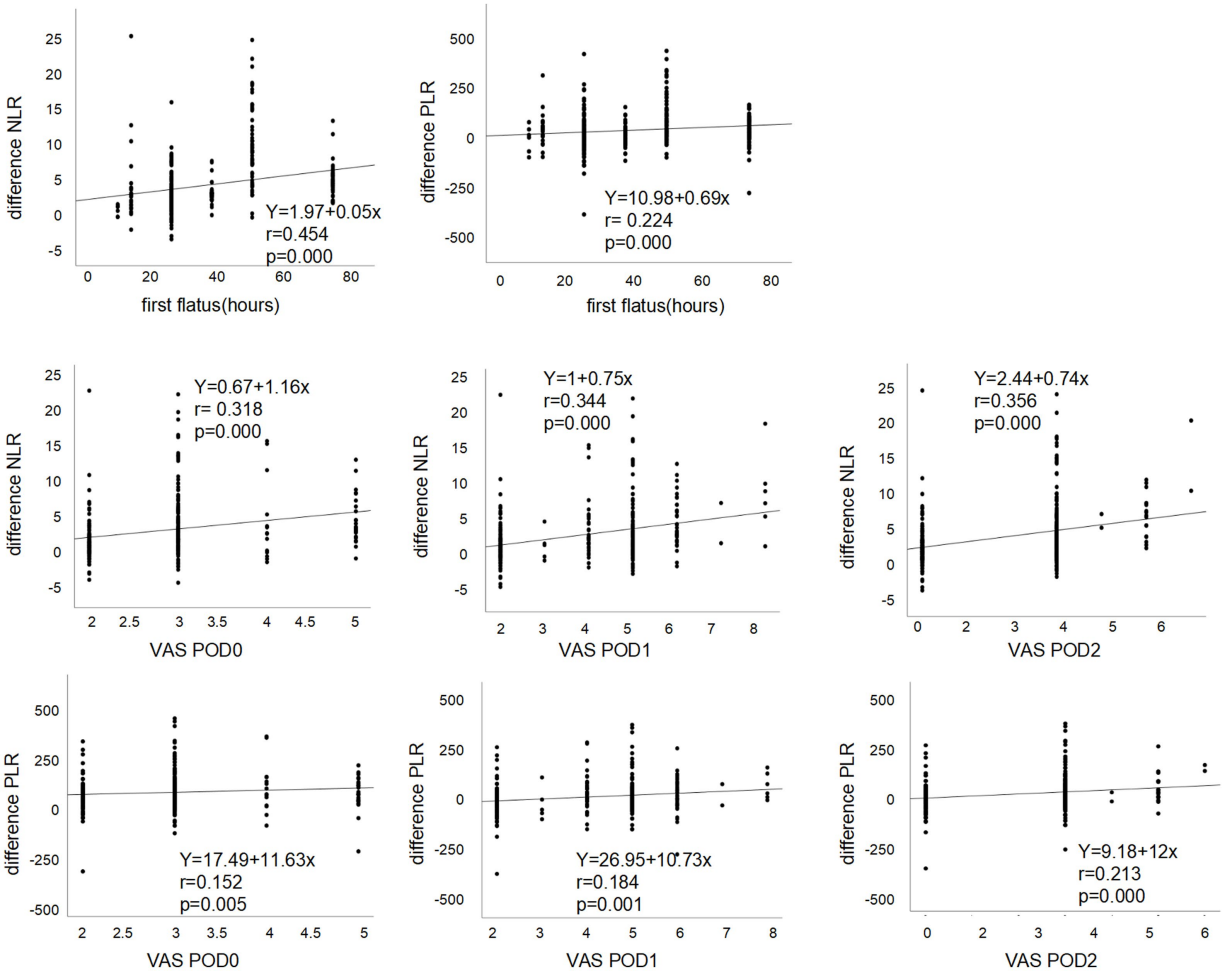
Linear regression and scatter diagram between first flatus time postoperatively, VAS score in POD0, POD1, and POD2 of patients following gynecologic surgery and the perioperative difference between neutrophil-to-lymphocyte ratio (NLR) and platelet-to-lymphocyte ratio (PLR).

We evaluated that the modulation of the ERAS pathway on the systemic inflammatory response (SIR), including the perioperative difference in the composite of blood, was evaluated, including WBC, neutrophils, lymphocytes, monocytes and platelets, NLR, PLR, and MLR in the ERAS and conventional groups. We identified that the difference between NLR and PLR preoperatively and postoperatively in enhanced recovery pathway patients significantly decreased compared to the conventional group (Figure 3). Moreover, linear regression analysis and scatter diagram shows that first flatus time postoperatively, VAS score in POD0, POD1, and POD2 of patients following gynecologic surgery is positively correlated to perioperative difference neutrophil-to-lymphocyte ratio (NLR) and platelet-to-lymphocyte ratio (PLR) (Table 4; Figure 4). There is no association between PONV and SIR (*p* = 0.108, 0.539, Table 4). Next, in order to figure out which element of the ERAS protocol has an impact on SIR, we evaluated each element of the ERAS protocol and identified that the perioperative difference between neutrophil-to-lymphocyte ratio (NLR) and platelet-to-lymphocyte ratio (PLR) is positively correlated with first sips of water time postoperatively, first semifluid diet time postoperatively, pelvic drain duration, and negatively correlated with out-of-bed time in PDO0 of patients following gynecologic surgery, not with elimination bowel preparation, IV fluid administration in POD0, and urinary catheter duration (Table 5).

**Table 4:** Correlation analysis between PONV, first flatus time postoperatively, VAS score in postoperative days POD0, POD1, and POD2 of patients following gynecologic surgery and the perioperative difference between neutrophil-to-lymphocyte ratio (NLR) and platelet-to-lymphocyte ratio (PLR).

SIR		PONV	First flatus	VAS POD0	VAS POD1	VAS POD2
Difference NLR	*r*	0.087	0.454	0.318	0.344	0.356
	*p*	0.108	0.000	0.000	0.000	0.000
Difference PLR	*r*	0.033	0.224	0.152	0.184	0.213
	*p*	0.539	0.000	0.005	0.001	0.000

**Table 5:** Correlation analysis between perioperative difference between neutrophil-to-lymphocyte ratio (NLR), platelet-to-lymphocyte ratio (PLR), and elements of ERAS protocol, including elimination bowel preparation time, IV fluid administration volume in postoperative day POD 0, first sips of water time postoperatively, first semifluid diet time postoperatively, urinary catheter duration, pelvic drain duration, and out of bed time in POD 0 of patients following gynecologic surgery.

Elements of ERAS protocol		Eliminate bowel preparation	IV fluid administration volume in POD0	First sips of water time postoperatively	First semifluid diet time postoperatively	Urinary catheter duration	Pelvic drain duration	Out of bed time in POD0
Difference NLR	*r*	−0.099	0.102	0.170	0.147	0.097	0.121	−0.100
	*p*	0.069	0.059	0.002	0.007	0.074	0.025	0.047
Difference PLR	*r*	−0.064	0.093	0.199	0.173	0.104	0.138	−0.150
	*p*	0.241	0.088	0.000	0.001	0.056	0.011	0.006

## Discussion

Enhanced recovery after surgery programs, which typically focus on minimizing preoperative stress and improving the response to postoperative stress, have grown substantially in modern surgical care. The systemic inflammatory response (SIR) is the direct manifestation of surgical stress. Although ERAS generates a reduced PONV, less pain, and shorter bowel recovery following gynecological surgery (29), evidence of the effect of ERAS protocols on SIR to the gynecological surgery is limited. No studies examined the impact of ERAS programs versus conventional perioperative care on the systemic inflammatory response (SIR) in gynecological surgery, making the interpretation of the inflammatory impact of ERAS protocols difficult. Instead of focusing on the length of hospital stay of patients and remarkable economic benefit, we instead focused on the modulation of ERAS protocols for the systemic inflammatory response to surgery. Consistent with our previous data in gynecological oncology surgery (30), here we first identified that ERAS protocols decrease the perioperative difference between PLR and NLR, alleviating the excessive inflammatory response status in patients with gynecological benign disease surgery.

Moreover, we found out NLR or PLR is positively correlated to pain score and first flatus time. The NLR or PLR has become a marker for gynecological surgical patients since it may directly affect pain and the return to normal bowel function. Analgesic regimens that minimize opioid demand, which may cause vomiting and intestinal dysfunction, are therefore key components of ERAS programs. It has been reported that local anesthesia techniques such as the transversus abdominis plane block (TAP) are successful in multiple surgical specialties and procedures (31, 32). Postoperative use of non-steroidal anti-inflammatory drugs (NSAIDs) has a good effect, which allows for more intense postoperative physical therapy and early mobilization, and results in lower pain scores (33, 34). Better pain control promotes more exercise, which stimulates faster bowel function recovery. Both faster bowel function recovery and less pain were seen in patients undergoing ERAS, and this is a consequence of a shorter systemic inflammatory status. Thus, here, we first demonstrate that the NLR or PLR could be used to assess the anesthetic effect and faster recovery of intestinal function.

Adherence to ERAS protocol improves SIR in patients undergoing gynecological surgery. Nevertheless, there is a lack of studies investigating a single ERAS item on systemic inflammatory responses. We needed to determine which individual interventions from ERAS contributed the most to the modulating systemic inflammatory response (SIR) markers. We demonstrated that the ERAS program, including early feeding, early ambulation after surgery, and shorter pelvic drain duration, was related to the perioperative difference between NLR and PLR. Early postoperative drinking and eating following gynecological surgery can reduce the systemic inflammatory response as part of an ERAS protocol. Meanwhile, within an enhanced recovery program following gynecological surgery, the more mobilization on postoperative day 0, the lower the NLR or PLR. The routine use of pelvic drains following gynecological surgery increases NLR and PLR. Therefore, early removal of pelvic drains can reduce the systemic inflammatory response as part of an ERAS protocol. In fact, enhanced protocols including early oral feeding and more mobilization lead to reduce SIR.

Although avoiding mechanical bowel preparation (MBP) has no adverse effect on postoperative complication rates, certain doctors and patients still feel anxious. Good education is necessary; Instead of bowel preparation, a clear liquid diet followed before surgery alleviates the anxiety of patients and doctors. There was no impact on surgical exposure by avoiding MBP following gynecological surgery. First, we identified that an MBP was not associated with the systemic inflammatory response as part of the ERAS protocol. Previous studies have shown that postoperative serum interleukin-6 is significantly lower following goal-directed therapy compared with conventional fluid management in colorectal surgery. However, our study found that NLR or PLR was not affected by intravenous (IV) fluid restriction in gynecological surgery. This finding raises the possibility that some of the components of ERAS programs provide little additional benefit to the SIR. In fact, postoperative C-reactive protein (CRP) in colorectal surgery was reported to be lower in laparoscopic surgery than in the open surgery group, regardless of perioperative care regimens (15, 35). However, there are limited data regarding clinical trials of individual components of ERAS protocols, which can lead to a reduction in the stress response following gynecological surgery.

Therefore, we demonstrated certain ERAS items, including early feeding postoperatively, early ambulation after surgery, and shorter pelvic drain duration, may attenuate system inflammation response, leading to a faster recovery of patients following gynecological surgery.

The strength of our study is the first clinical trial revealing the relationship between the component of the ERAS program and systemic inflammatory response in gynecological surgery. This study could help clinicians understand the effectiveness of enhanced recovery protocols in the modulating systemic inflammatory response. The benefits of an attenuated systemic inflammatory response could be a key to enhancing recovery and can be a protective feature for patients. Meanwhile, the limitation of this study is that more objective indicators are needed to reflect the effects of ERAS, and more research needs to be carried out in the future for a better evaluation of the molecular mechanism of ERAS on the systemic inflammatory response.

## Conclusion

The enhanced recovery after surgery protocol provides faster recovery, less postoperative pain, decreased incidence of postoperative nausea and vomiting, and shorter hospital stays after gynecological surgery, which is a consequence of improved inflammatory status. Indeed, alleviated NLR or PLR could be a systemic inflammatory response predictor of ERAS programs’ success in gynecological surgery. Moreover, we identified that certain ERAS items, including early feeding postoperatively, early ambulation after surgery, and shorter pelvic drain duration, applied to different types of gynecological surgery have a role in alleviating systemic inflammatory response.

## Data availability statement

The raw data supporting the conclusions of this article will be made available by the authors, without undue reservation.

## Ethics statement

The studies involving human participants were reviewed and approved by the Ethics Committee of Qilu Hospital (scientific research review N0.2018-141). The patients/participants provided their written informed consent to participate in this study.

## Author contributions

NX and JP carried out the study, analyzed the data, and wrote the manuscript. YH and HW were involved in the study design, data management, and study analysis. All authors have made a great contribution to the manuscript, read, and approved the final manuscript.

## Funding

This study was supported by grants from the National Natural Science Foundation of China (Project No. 82272781), Shandong Medical Association Clinical Scientific Research Fund - Qilu Special project (YXH2022ZX02144), and Beijing Xisike Clinical Oncology Research Foundation (Y-MSDPU2022-0223).

## Conflict of interest

The authors declare that the research was conducted in the absence of any commercial or financial relationships that could be construed as a potential conflict of interest.

## Publisher’s note

All claims expressed in this article are solely those of the authors and do not necessarily represent those of their affiliated organizations, or those of the publisher, the editors and the reviewers. Any product that may be evaluated in this article, or claim that may be made by its manufacturer, is not guaranteed or endorsed by the publisher.
